# Introducing the participant-generated experience and satisfaction (PaGES) index: a novel, longitudinal mixed-methods evaluation tool

**DOI:** 10.1186/s12874-023-02016-1

**Published:** 2023-09-28

**Authors:** Andrew Symon, Kate Lightly, Rachel Howard, Shuchita Mundle, Brian Faragher, Molly Hanley, Jill Durocher, Beverly Winikoff, Andrew Weeks

**Affiliations:** 1 Mother and Infant Research Unit, University of Dundee, 11 Airlie Place, Dundee, DD1 4HJ UK; 2 Clinical Research Fellow, University of Liverpool, Liverpool Women’s Hospital, Crown Street, Liverpool, L8 7SS UK; 3 Present Address: Medical Student, University of Liverpool, Liverpool, L69 3BX UK; 4 Department of Obstetrics and Gynecology, All India Institute of Medical Sciences, Nagpur, India; 5 Liverpool School of Tropical Medicine, Pembroke Place, Liverpool, L3 5QA UK; 6 Gynuity Health Projects (GHP), MOLI Trial Manager, 220 East 42nd Street, New York, NY 10017 USA; 7 Sanyu Research Department, University of Liverpool, Liverpool Women’s Hospital, Crown Street, Liverpool, L8 7SS UK

**Keywords:** Participant, Experience, Satisfaction, Patient-generated, Qualitative research, Quantitative research, Instrument, Birth

## Abstract

**Background:**

Patient-Reported Outcomes or Experience Measures (PROMS / PREMS) are routinely used in clinical studies to assess participants’ views and experiences of trial interventions and related quality of life. Purely quantitative approaches lack the necessary detail and flexibility to understand the real-world impact of study interventions on participants, according to their own priorities. Conversely, purely qualitative assessments are time consuming and usually restricted to a small, possibly unrepresentative, sub-sample. This paper, which reports a pilot study within a randomised controlled trial of induction of labour, reports the feasibility, and acceptability of the Participant-Generated Experience and Satisfaction (PaGES) Index, a new mixed qualitative / quantitative PREM tool.

**Methods:**

The single-sheet PaGES Index was completed by hypertensive pregnant women in two hospitals in Nagpur, India before and after taking part in the ‘Misoprostol or Oxytocin for Labour Induction’ (MOLI) randomised controlled trial. Participants recorded aspects of the impending birth they considered most important, and then ranked them. After the birth, participants completed the PaGES Index again, this time also scoring their satisfaction with each item. Forms were completed on paper in the local language or in English, supported by Research Assistants. Following translation (when needed), responses were uploaded to a REDCap database, coded in Excel and analysed thematically. A formal qualitative evaluation (qMOLI) was also conducted to obtain stakeholder perspectives of the PaGES Index and the wider trial. Semi-structured interviews were conducted with participants, and focus groups with researchers and clinicians. Data were managed using NVivo 12 software and analysed using the framework approach.

**Results:**

Participants and researchers found the PaGES Index easy to complete and administer; mothers valued the opportunity to speak about their experience. Qualitative analysis of the initial 68 PaGES Index responses identified areas of commonality and difference among participants and also when comparing antenatal and postnatal responses. Theme citations and associated comments scores were fairly stable before and after the birth. The qMOLI phase, comprising 53 one-to-one interviews with participants and eight focus groups involving 83 researchers and clinicians, provided support that the PaGES Index was an acceptable and even helpful means of capturing participant perspectives.

**Conclusions:**

Subjective participant experiences are an important aspect of clinical trials. The PaGES Index was found to be a feasible and acceptable measure that unites qualitative research’s explanatory power with the comparative power of quantitative designs. It also offers the opportunity to conduct a before-and-after evaluation, allowing researchers to examine the expectations and actual experiences of all clinical trial participants, not just a small sub-sample. This study also shows that, with appropriate research assistant input, the PaGES Index can be used in different languages by participants with varying literacy levels.

**Trial registration:**

Clinical Trials.gov (21/11/2018) (NCT03749902).

**Supplementary Information:**

The online version contains supplementary material available at 10.1186/s12874-023-02016-1.

## Background

The inclusion of Patient-Reported Outcomes and Experience Measures (PROMS / PREMS) in larger studies [1] [2] reflects the value of including participants’ voices in research. Such tools can shed light and context on the ‘hard’ outcomes that will continue to be the main focus of most randomised controlled trials (RCTs) [3] and they have now been used in a range of disciplines in an attempt to “seek to ascertain patients’ views of their symptoms, their functional status, and their health-related quality of life” [4]. Such measures can address satisfaction and subjective experiences – for example of pain or of care provider performance [5] including obstetric racism [6]. These evaluations acknowledge that research studies may not capture all relevant outcomes and experiences, even when Patient and Public Involvement (PPI) is prioritised throughout the design and implementation of the research.

Using a PREM offers the chance to evaluate a patient’s experience of the process of care rather than feelings about the clinical outcome, and most focus on dimensions of care known to be important to patients [7]. Ideally, a short instrument will “capture patients’ perceptions of the service they had experienced with minimal effort, [and] give rapid feedback to all stakeholders in a way that [is] comparable, scalable and economical” [8] (p113). PREMs (like PROMS) can be administered to a larger group than might be included in a qualitative sub-study within a trial, but they must still reflect what is most important to the participants. However, despite an eclectic range of pregnancy and birth-related measurements focusing on both pathological and salutogenic (wellbeing-related) outcomes [9, 10], there is still no consensus about which outcomes or experiences are most relevant since these are likely to vary from one study to another. Preparatory qualitative work can identify the most likely variables for a particular study, but this takes time and cannot guarantee that all such relevant variables will be identified.

An alternative approach is to use a PREM which identifies and evaluates a patient’s subjective experience. The Mother-Generated Index (MGI) [11], for example, is a subjectively-derived tool that combines a brief qualitative evaluation of personal experience with a quantitative element. We modified the MGI for use in a longitudinal pilot study within a randomised controlled trial involving pregnant women who were being induced (MOLI [NCT03749902] [12]). The aim of this pilot study was to assess the feasibility and acceptability of this novel participant-generated experience measure, originally called the Mother-Generated Birth Satisfaction Index (MGBSI). To assess ‘feasibility’ we evaluated, within the study team, the practicalities of including this PREM-based aspect within the wider study, specifically whether women could comprehend and successfully complete the questionnaire, and whether useful data could be extracted from it. ‘Acceptability’ was gauged by the ease or otherwise with which participants engaged with this aspect of the study and were able to complete the survey form and was assessed during qualitative interviews with women and researchers. During a pause in study recruitment due to the COVID pandemic, we re-named the tool the Participant-Generated Experience and Satisfaction (PaGES) Index in view of the evident potential generic role of this tool in future studies. The aim of this paper, then, is to report on the feasibility and acceptability of the PaGES Index as a novel PREM.

### Induction of labour

Since the 1970s, induction of labour (IOL) in childbirth has increased steadily [13]. Most research into IOL has focused on the various methods used and their management, with little research focusing on women’s experience and satisfaction [14]. Alfirevic et al’s systematic review of IOL found that “less than 5% of the studies … reported data relating to maternal satisfaction with the induction process” [15] (p.60). Due to the potential for poor satisfaction associated with this intervention (e.g. pain, duration of the process) [16, 17], we set out to evaluate participants’ anticipated and actual experiences of induction of labour. This PREM study was conducted alongside a multi-centre parallel, superiority, open-label randomised trial in two publicly funded hospitals in India (the MOLI study [12]). This RCT compared oral misoprostol and intravenous oxytocin to augment labour. Women who had received misoprostol to induce labour, and who were deemed to need further induction agents after a stated time, were eligible to enter the MOLI trial. The results of the main RCT will be published elsewhere.

## Methods

This was a pilot study, cross-sectional in design, conducted within the main MOLI randomised trial to assess the feasibility and acceptability of the PaGES Index. The pilot study took place in two public hospitals in Nagpur, India. While 1,000 women were recruited to the MOLI study (helped when a third site came on stream in 2021), this pilot study involved only the first 68 participants, irrespective of randomisation arm. As part of the pilot study, a coding framework was created and analysed. This informed the data analysis plan for the main dataset.

In order to assist those interested in adopting a participant-generated PREM approach, we describe the forms and the process of this pilot study, including the analysis, in some detail. The assessment of feasibility and acceptability comprised two elements. The first involved administering the PaGES Index before and after the birth to MOLI study participants. The second utilised data from an alongside qualitative study evaluating the main MOLI trial and involved a series of one-to-one interviews with study participants, and focus groups with Research Assistants (RAs) and clinicians. This second element is known as the qMOLI study.

The PaGES Index study participants were women undergoing labour induction because of pre-eclampsia or hypertension at three public hospitals in Nagpur, India. The first PaGES Index was completed after recruitment to the MOLI RCT but before randomisation. Inclusion criteria included being 18 years and above, being able to give consent, having a live singleton fetus, and having no history of allergy to misoprostol or previous history of caesarean section ([12]). Women in the MOLI study were asked to complete the PaGES Index on recruitment, and again postnatally. They completed a second PaGES Index form postnatally. Follow-up was 100%, eliminating loss to follow-up bias. As the aim of this paper is to report the feasibility and acceptability of the PaGES Index, and not to report the study results per se, we do not report the significance of participants’ socio-demographic and other characteristics. The RAs at the participating sites were trained in qualitative research methods making them competent to conduct the PaGES Index interviews and record the participants’ responses without bias or influence.

Women were recruited to the qMOLI study, according to a pre-defined sampling frame aiming to incorporate different groups of women from the study; differing mode of births, parities, socioeconomic status, recruiting site. Clinicians involved in screening, recruiting, randomising, and consenting participants to the MOLI RCT were also included in the qMOLI qualitative evaluation phase.

### The PaGES Index

**Fig. 1:**
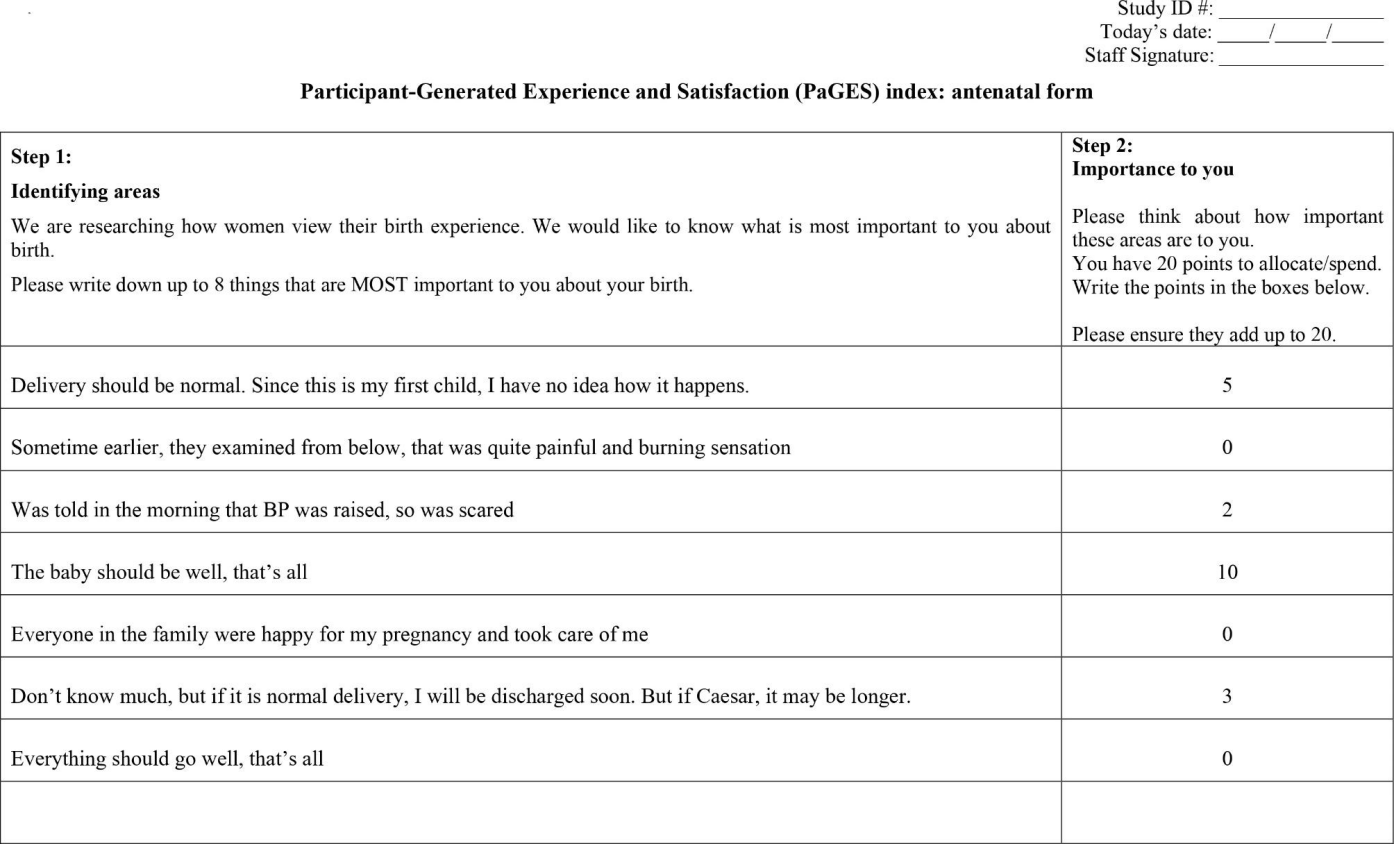
Example of completed antenatal PaGES Index form

**Fig. 2:**
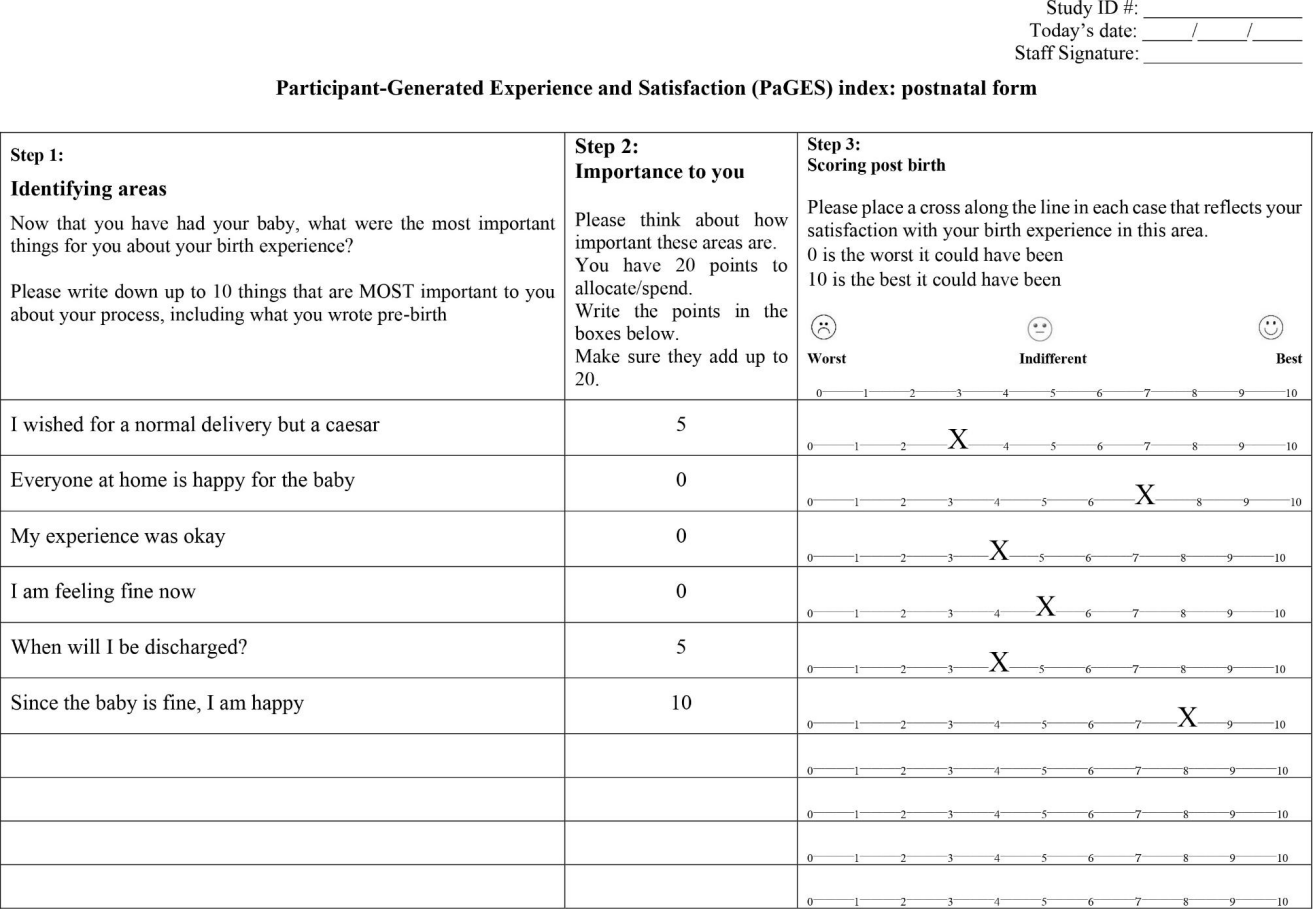
Example of completed postnatal PaGES Index form

The Participant-Generated Experience and Satisfaction (PaGES) Index is a modified version of the Mother-Generated Index (MGI) [11]. Templates for the antenatal and postnatal PaGES Index forms were generated (Figs. 1 and 2) and used from the start of the randomised trial in January 2020. Both antenatal and postnatal evaluations comprise Step 1 (‘Identifying areas’) and Step 2 (‘Importance to you’). The scoring is only possible following the event and this is added as postnatal Step 3 (‘Scoring post birth’).

Having entered the MOLI trial, participants were asked to complete the PaGES form by writing down or telling the RA “up to 8 things that are MOST important to you about your birth”; these statements are referred to as ‘comments’. Because it was known that the client population had varying literacy levels, participants were given the option of completing the form themselves or dictating their responses to the RA who would then complete the form. Comments were recorded verbatim in the woman’s preferred language (English, Hindi or Marathi) on the paper Case Report Form (CRF). The participant then distributed 20 ‘spending beans’ across her comments to demonstrate the relative importance of each to her at that time. The RAs made it clear that not all comments had to be allocated a bean. Finally, the RA checked back with the participants to ensure the comments and their importance were recorded correctly. Appropriately skilled bi-lingual researchers translated the forms into English in an electronic format.

The postnatal PaGES Index was usually completed within 24–48 hours of the birth (range 1–6 days). Where possible, this was conducted by the same RA who had assisted with the antenatal PaGES Index. Postnatally, the participant identified up to ten things that were MOST important to her about her birth experience, relatively ranked these using the 20 beans, and then scored each comment out of ten to indicate experience and satisfaction (range 0 [worst] to 10 [best]) [See Supplementary Box 1 for explanation of process]. Two additional comments were allowed postnatally because we wanted to allow more space to comment on the experience of or satisfaction with the intervention. The women were not shown their equivalent antenatal form when completing the postnatal assessment. Completing the forms took between five and ten minutes. Data collection took place between January and March 2020 at two study sites. However, the onset of the Covid pandemic resulted in a 7-month pause in recruitment. With the uncertainty about when the MOLI study would be able to restart (including when the third proposed study site would be available) it was decided to treat the data collected from these 68 women during this initial phase as pilot data. We concede there was an element of enforced pragmatism about the sample size and the timing, but this was unavoidable given the circumstances of the Covid pandemic.

A senior research team member cross-checked the translated forms to ensure accuracy. A second expert translator reviewed errors in translation or disagreements. The anonymised translated text and data were then entered into an online REDCap database [https://www.project-redcap.org]) and exported into Excel for coding.

### Qualitative analysis of PaGES Index

The comments were inductively and independently coded using line-by-line thematic coding [18] by three researchers (RH, AS, KL). Inter-rater discussions confirmed when coding refinements were needed and the subsequent aggregation of codes into themes and then over-arching themes. An example of first-level coding discussions included the eventual agreement that the comments “My baby should be safe and deliver in a good way”, “For my baby I will bear a pain, whatever …” and “The baby should be fine and healthy” formed the code “Baby is the priority”. Where there was uncertainty, or where inter-rater discussions did not resolve the disagreement, we requested arbitration by a study partner in India (SM) who referred back to the original forms, particularly those in Hindi/Marathi, to ensure the meaning had been adequately captured. The wider research team was also consulted on the developing analysis. A revised coding framework was discussed and finalised before its application to the whole pilot (n = 68) data set.

### Quantitative analysis (PaGES Index)

Quantitative analyses were confined to the overarching themes given the numbers in this pilot study. The numbers citing each theme antenatally and/or postnatally are summarised as frequency counts and proportions (with 95% confidence intervals [CI]). Differences in the antenatal / postnatal counts were evaluated using the McNemar test for paired frequency counts.

As many mothers cited more than one important issue within the same theme, the total frequency with which each theme was cited antenatally and postnatally is summarised, along with the total numbers of times (a) no beans and (b) one or more beans were allocated to a particular comment. The percentage of comments attracting one or more beans is reported along with the difference in this percentage between the two assessment times (with 95% CI).

The total numbers of beans allocated to each theme antenatally and postnatally are reported as means and standard deviations (SD). The differences between the two assessment times are summarised as rate ratios (with 95% CI), estimated using generalised linear models assuming a Poisson family and logarithmic link, and robust standard errors to adjust for over-dispersion and clustering of scores within subjects. Separate analyses were conducted using all counts (zero and non-zero) and using just the non-zero counts.

Satisfaction scores are summarised using means and SD. The relationships between these scores and the numbers of beans allocated antenatally and postnatally for each theme, as well as the differences in bean allocations at the two times (postnatal minus antenatal), were estimated using generalised linear models assuming a Normal (Gaussian) family and identity link, and with robust standard errors to adjust for clustering of observations within subjects. Again, separate analyses were conducted using all counts (zero and non-zero) and using just the non-zero counts.

Finally, the sensitivity of the satisfaction scale to detect differences between sub-groups was explored. A hybrid measure, calculated as ‘(satisfaction score + 1) * bean allocation’ was also explored; since this measure was very positively skewed, it was transformed to natural logarithms for analysis. These are exploratory analyses given the relatively small numbers in this pilot study. The scores from both measures were reported as means and SD. Differences between groups were evaluated using generalised linear models assuming a Gaussian family and identity link.

### qMOLI phase - formal qualitative evaluation

A formal qualitative element of the whole MOLI RCT was undertaken to assess the priorities, experiences, and acceptability of IOL for women in the MOLI study. It also covered the researchers’ and clinicians’ views of the feasibility, usability, acceptability, and barriers to implementation of the study. The qMOLI process and results reported here date from September to December 2021, when Covid restrictions had been lifted; thus while the women taking part in the qMOLI interviews had all completed the PaGES Index, and had given birth in the same two hospitals, they are not the same women whose PaGES responses are reported here. The interviews included a review of the PaGES Index administration, and this is reported here. Women were eligible for inclusion in the qMOLI study if they were recruited to the MOLI RCT and provided informed consent. Research Assistants (RAs) who administered the PaGES Index forms were interviewed; in addition, clinicians involved in screening, recruiting, randomising, and consenting participants to MOLI RCT were also eligible. Full recruitment details can be found in the registered qMOLI protocol at ClinicalTrials.gov (NCT04037683).

Before recruitment, a sampling frame was outlined to specify key characteristics such as parity, mode of birth, induction methods for women and cadres of staff from the recruitment team. One-to-one interviews were conducted in the language of the patient’s choice (Hindi/Marathi), by a trained RA with a background in clinical research and Homeopathic medicine; these continued until data saturation was met. Two focus groups were conducted pre-trial in 2019 and a further six mid-trial in September – December 2021 by a senior qualitative researcher and clinician. Interview schedules were devised by the research group and reviewed throughout the project. FGDs were planned to capture data both before and during trial, in order to understand anticipated versus lived experiences amongst the research team.

Interviews and focus group discussions were audio recorded and transcribed verbatim, including any observations noted. Interviews were translated into English by the interviewer and reviewed by a research team member. Researchers familiarised themselves with the data through post-interview meetings, note-keeping, re-reading, and detailed memo writing. Codes were generated using a primarily inductive approach through open coding of the data. Working independently, two researchers (KL and CK) initially devised separate coding frameworks. These were then merged through consensus. The wider trial management group reviewed a selection of transcripts (interview 16, 21 and focus group 1) before finalising the initial coding framework. Codes were clustered into categories with “other” categories to avoid missing important data.

NVivo 12 software was used to code to the analytical framework before charting the data into matrices using summaries and illustrative quotes (KL, MH, LH). The data were thoroughly reviewed for common patterns to generate overall themes. Any differences in the data were examined for causality, and comparisons were explored between the different groups.

### Ethics

The MOLI study was approved by the Institutional Ethics Committees at Government Medical College Nagpur (1724 EC/Pharmac/GMC/NGP), Spandan Heart Institute and Research Center, Mahatma Gandhi Institute of Medical Sciences (MGIMS/IEC/OBGY/96/2020) and Liverpool (Ref. 4691). The qMOLI study (NCT04037683) was approved by the Research Ethics Committees in Nagpur and Liverpool (Ref. 1756). This trial is sponsored by the University of Liverpool (UoL001374). The study is registered with Clinical Trials.gov (NCT03749902) and Clinical Trial Registry, India (CTRI/2O W I A4l 0 I 8527).

## Results

### The PaGES Index

All women who were recruited to the main MOLI trial also completed both antenatal and postnatal PaGES Index forms. The first 68 to do so comprised this pilot study (see Table S1 for basic participant characteristics):

Since the aim of this paper is to report on the feasibility and acceptability of the PaGES Index, results are presented here only to indicate how the data can be managed and to illustrate the potential of this kind of PREM approach.

In Figs. 1 and 2 the Step 2 points spent between the participants’ comments are illustrative of relative importance. The Step 3 scoring in Fig. 2 produces a PaGES Index score of 5.2 ([3 + 7 + 4 + 5 + 4 + 8] / 6 = 5.2). There were a few omissions in the postnatal forms: five of the 68 women did not allocate a Step 2 score to at least one comment, and a further six did not complete Step 3.

The antenatal qualitative analysis produced 24 codes, 14 of which also emerged postnatally, along with a further 14 postnatal codes. Codes were aggregated to form 14 antenatal and 13 postnatal themes (eleven of which were shared) and in turn these themes were grouped to form six overarching themes **(**Table 1**)**.

**Table 1:** Antenatal and postnatal overarching themes, themes, codes

Overarching Theme 1: Perspectives on the birth			
Themes	Antenatal code	Themes	Postnatal code
“Be good”	Delivery should be good / safe	“Be good”	
	Everything should go well		Everything was good
Mode of birth	Prefer NVD	Mode of birth	Satisfaction with mode of birth - NVD
			Dissatisfaction with mode of birth - NVD
	Prefer CS		Satisfaction with mode of birth - CS
			Dissatisfaction with mode of birth - CS
	Prefer quick delivery		Prefer quick delivery
	No mode of birth preferences		Equivocal re mode of birth
	Reason for CS		Reason for CS
Pain	Scared / fear / afraid due to pain	Pain	
	Bear the pain		Pain during birth
			Postnatal pain
	General pain comments		General pain comments
Anticipating labour and birth	Knowledge	Reviewing labour and birth	Knowledge
	Preparedness		
	Medication		Medication
			Was scared / fear / afraid / worried / anxious prior to birth
		“Traas”	Difficulty / trouble
		Treatment by staff	Staff are good / take care
**Overarching Theme 2: Perspectives on the baby**			
Baby should “be good”	Baby is the priority	Baby should “be good”	Baby is the priority
			Because baby is good, I am good
			Concerns / worry about baby pre delivery
			Concerns / worry about baby post delivery
Gender	Gender of baby	Gender	Gender of baby
	Family and baby’s gender		Family and baby’s gender
Breastfeeding	Breastfeeding	Breastfeeding	Breastfeeding
**Overarching Theme 3 - physical condition**			
Blood pressure & Physical Symptoms	Blood pressure & other physical symptoms	Blood pressure & physical symptoms	Blood pressure & other physical symptoms
**Overarching Theme 4 - psychological condition**			
Happy	Happy	Happy	Happy
Scared / fear / afraid, worried / anxious	Worried / anxious / scared / fear / afraid		
No worries	No worries	No worries	No worries / difficulty
**Overarching Theme 5 - Family**			
Family influences	Family is good / happy / take care		Family is good / happy / take care
**Overarching Theme 6 - Looking to the Future**			
Looking to the future	Looking to the future	Looking to the future	Looking to the future

24 Antenatal codes (14 also PN) 28 Postnatal codes (14 also AN)

These themes and codes demonstrate how participants in this study broadly shared similar views and experiences; and yet no two forms were identical. Individual experience, therefore, can be tracked. The first over-arching theme (‘Perspectives on the Birth’) is explored in more detail in Table 2, with the remaining five over-arching themes (2 - Perspectives on the Baby; 3 - Physical Condition; 4 - Psychological Condition; 5 - Family; 6 - Looking to the future reported in supplementary files.

**Table 2:** Overarching theme 1 - perspectives on the birth: results of antenatal and postnatal inductive coding

Antenatal			Postnatal			
Themes	Antenatal code	To summarise comments such as:	Themes	Postnatal code	To summarise comments such as:	
“Be good”	**Delivery should be good / safe**	The delivery itself should “be good,” go well, be safe, includes thoughts of worry or anxiety surrounding the delivery	“Be good”			
	**Everything should go well**	Includes mother and both baby and mother. Everything, all should be “good,” go well, be safe. Baby and mum should be good / healthy / safe		**Everything was good**	Everything went well, delivery was good / safe, baby and I are healthy	
Mode of birth	**Prefer NVD**	Wants / prefers NVD. NVD is good, NVD is better. Don’t want CS.	Mode of birth	**Satisfaction with mode of birth – NVD**	Happy had NVD, prefer normal, want normal	
				**Dissatisfaction with mode of birth – NVD**	Wanted CS	
	**Prefer CS**	Wants / prefers / would choose CS. Don’t want NVD		**Satisfaction with mode of birth – CS**	Happy had CS, wanted CS, CS is better	
				**Dissatisfaction with mode of birth – CS**	It would have been better if I had a normal delivery; wanted NVD, had CS	
	**Prefer quick delivery**	Wants labour and delivery to be quick / fast. Includes 2 statements of “quick and normal delivery”		**Prefer quick delivery**	Long labour / better if quicker, references to time of delivery	
	**No mode of birth preferences**	No mode of birth preferences, happy for normal birth or CS		**Equivocal re mode of birth**	OK with CS, if better for baby	
	**Reason for CS**	Several statements about why CS may be done, not including preferences etc.		**Reason for CS**	Several statements about why CS was done, not including preferences etc., includes doctors tried for NVD but CS was done	
Pain	**Scared / fear / afraid due to pain**		Pain			
	**Bear the pain**	I cannot tolerate pain, I cannot bear the pain, will try to bear the pain, I am ready to bear the pain, will bear the pain		**Pain during birth**	Pain during delivery includes lots of pain	
				**Postnatal pain**	Includes pain now (postnatal)	
	**General pain comments**	Pain general comments		**General pain comments**	Pain general comment, includes comments of small pain during or before birth	
Anticipating labour and birth	**Knowledge**	I don’t know much delivery / pain / both. Include both what women do and do not know. Includes knowledge of problems associated with delivery types	Anticipating labour and birth	**Knowledge**	I don’t know much about delivery / pain / both. Include both what women do and do not know. Includes knowledge of problems associated with delivery types	
	**Preparedness**	Anticipating labour and birth, focus on preparedness, ready to do whatever has to be done (Tx, Mx, Dr), excluding pain				
	**Medication**	Opinions regarding the use of medication in their care		**Medication**	References to medication	
				**Was scared / fear / afraid / worried / anxious prior to birth**	Comments regarding worry, anxiety and fear expressed in the past tense prior to birth	
			“Traas”	**Difficulty / trouble**	Comments using the words difficulty or trouble referring to the birth “traas”	
			Treatment by staff	**Staff are good / take care**	References to the treatment of staff	

Key: NVD - Normal Vaginal Delivery CS - Caesarean Section Tx - Treatment Mx - Medication Dr - Doctor

We are not reporting study findings per se but presenting the PaGES Index for consideration for inclusion in other trials. Table 2 (and Tables S2 – S3) show how the coding structure works: examples of comments are given together with their allocated code; the aggregation of these codes produces themes; and these in turn, when aggregated, produce over-arching themes.

#### Analysis of bean counts

The numbers citing each of the six overarching themes are summarised in Table 3. In general terms, each theme was cited by fewer mothers postnatally than antenatally, with two exceptions. “Perspectives on the baby” was cited essentially equally in both assessments, while “looking to the future” was cited just once antenatally but by almost a quarter of mothers postnatally. This was statistically highly significant (p < 0.001) despite the small sample.

**Table 3:** Numbers of mothers recording each overarching theme (antenatal vs. postnatal)

Overarching theme:	n (%) [95% confidence interval]		difference n (%) [95% CI]
	antenatal	postnatal	
**Perspectives on the birth**	68 (100) [94.7–100]	65 (95.6) [87.6–99.1]	-3 (-4.4) [-10.8–1.9]
**Perspectives on the baby**	56 (82.4) [71.2–90.5]	57 (83.8) [72.9–91.6]	+1 (1.4) [-11.2–14.1]
**Physical condition**	22 (32.4) [21.5–44.8]	15 (22.1) [12.9–33.8]	-7 (-10.3) [-26.0–5.4]
**Psychological condition**	44 (64.7) [52.2–75.9]	38 (55.9) [43.3–67.9]	-6 (-8.8) [-26.5–8.8]
**Family**	44 (64.7) [52.2–75.9]	39 (57.4) [44.8–69.3]	-5 (7.3) [-24.8–10.1]
**Looking to the future**	1 (1.5) [0.04–7.9]	16 (23.5) [14.1–35.4]	+15 (22.0) [12.3–45.4]

With such a dataset it can be seen that the association of socio-demographic or clinical trial characteristics with particular types of comment or the average scores given to certain comments can be made. Illustrative analyses covering the number of times each of the six overarching themes were cited, the mean bean allocation counts for each theme, the mean postnatal scores for each theme and the mean satisfaction and hybrid ‘satisfaction*bean count’ scores are described in the supplementary file ‘Additional quantitative analysis’.

However, as previously stated, these analyses are merely illustrative of the potential of the PaGES Index.

### The qMOLI phase

Fifty-three semi-structured interviews were conducted with 45 women (19 before and 34 after induction; 8 before and after). Women represented a range of backgrounds, including socioeconomic status and obstetric history. Postnatal interviews were conducted between days one and six and included women from both study arms. In addition, eight focus groups were held with 83 clinicians and RAs either before trial commencement (n = 2) or during trial delivery (n = 6). Only the PaGES Index aspects of the qMOLI dataset are reported here.

Most women could recall using the tool, and their experiences were positive. They explained how to use the tool and especially the ‘spending beans’ element:

“I have (given) good marks to whatever good feelings were there” (P7).

Some women shared that this was “a new experience” (P47), “felt nice” (P14) or was “like a game” (P43).

“You asked me these questions, and I liked that a lot.” (P29).

Some were indifferent to the use of the tool, with short neutral answers, such as “yes, filled” (P5). There were only occasional negative reports, with one woman describing “I didn’t pay attention” (P23) or that her “feet tingled” (P16), due to the time taken. However, some shared concerns about confidentiality and sharing information that may cause them problems:

“How can I talk freely?” (P52).

“That time I was thinking that something I said will go against my family.” (P21).

Women reported that the tool was easy to complete:

“It was easy, that’s why I could do that.” (P18).

Most reported that they shared all of their feelings and emotions when completing the tool. For example,

“Whatever was in my mind, I told.” (P12).

Several were pleased to be doing something that could benefit other women in the future by taking part in this aspect of the study:

“Whatever my information, my feelings are there. That will be useful for others.” (P21).

When asked specifically if any aspects of the tool could be improved, no participant recommended any changes.

“Everything was there in that…I don’t feel any need for an improvement in that.” (P43).

The clinical staff were largely unaware of the use of the tool; however, the RAs who administered the tool gave fascinating insights into its role. They described how administering it made women feel good and described how the women enjoyed the way it made them feel.

“They feel that for nine months, no one asked me. After coming here, someone is asking me what my feeling is.” Research Associate, (FGD 7)

The Research Associates raised no concerns about the role and utility of the tool.

## Discussion

Aside from the White Ribbon Alliance’s survey in over 100 countries, including India, which tried to establish *What Women Want* [19], there has been limited research into what is important to Indian women about childbirth and what could be done to improve their birth experiences [20]. This paper, in reporting the incorporation of the PaGES Index, a participant-driven PREM, in an RCT of induction of labour in India, helps to address this deficit.

The PaGES Index combines qualitative and quantitative evaluations and can be used both before and after an intervention to evaluate experience and satisfaction within and between groups. It can also be used as a single-point three-step instrument, like the Mother-Generated Index [11] (MGI) on which it is based. Such tools offer flexibility: the MGI’s feasibility, acceptibilty, reliability and validity have been established in observational and before-and-after studies within several linguistic and cultural groups in twelve countries to date, including India [21]. It has also been used within an RCT in the UK [22] and in both the antenatal and postnatal periods [23–25].

Subjective evaluations have been criticised because of a lack of standardisation and of reliability data [26]. The link between patient satisfaction and health care or health care quality and outcomes has also been claimed to be unclear or even tenuous [27, 28]. However, we found in this study that the PaGES Index goes beyond traditional satisfaction measurements. Traditional satisfaction measures generally involve binary responses or the scoring of pre-written questionnaires. The PaGES Index adds granularity as it allows participants to use their own words to describe what is important for them in a relatively open-ended format. All women recruited to the MOLI trial agreed to complete the PaGES Index form antenatally. While all these women also agreed to complete the postnatal follow-up, we concede that a small number did not complete all of the postnatal form accurately. As a learning point, that is something we need to take on board. Nevertheless, overall we feel that this is a feasible and acceptable before-and-after measure, and moreover one that unites the explanatory power of qualitative research with the comparative power of quantitative designs. This study also demonstrated that it can be used successfully in different languages and with people of varying proficiency in reading skills. For this study we asked participants to allocate ‘spending beans’ between their comments, a stratagem borrowed from a Brazilian MGI study in which participants had struggled to conceptualise ‘spending points’ (the originally intended – and correct - term) but readily understood how to distribute a daily food item among their comments [29].

The number of mothers in this pilot phase of the MOLI study was too small to support inter-group statistical comparisons. The analyses and findings we have presented are therefore illustrative rather than formal. They are designed to show the instrument’s potential scope rather than to report the results of a particular study. However, while the mean bean count scores varied considerably (consistent with the theoretical expectation of a statistically over-dispersed Poisson distribution), with proper consideration of statistical power and sample size requirements and appropriate selection of statistical models, the bean count scores should be able to detect small but clinically important differences between groups. The PaGES Index, therefore, has the potential to provide a sufficiently sensitive instrument for use in clinical trials. Perhaps most obviously, this could be used to explore possible differences in experience between trial arms.

Despite most PROMs / PREMs being developed through a lengthy process of consultation and trial [30] these processes happen within specified contexts. Given that each clinical and trial context is different, there is always the possibility that something important may have been missed. The benefit of the PaGES Index, as with the Mother-Generated Index [11] and the original Patient-Generated Index (PGI) which inspired it [31], is that it includes what patients or research participants consider most important, and allows these to be compared before and after a specified intervention. This is a significant feature in terms of increasing the subjective input of the end-user in either clinical care or research.

The PaGES Index allows for the participant-identified similarities and differences in the before and after evaluation to be reviewed. From a qualitative perspective, a simple description can be made of what was said before and after, with areas of overlap and difference examined. Given the fundamental nature of the intervention in this study (induction of labour and childbirth) it is not surprising that the overarching themes were very similar antenatally and postnatally, although the underlying comments varied to a greater or lesser extent. This allows for a comparison to be made between anticipated and actual experience on two levels: what participants say is important to them and how they rank these.

Timing of administration is a critical feature. In this study women completed the PaGES Index at relatively short notice not long before the birth, and again within days of the birth. The degree of similarity in the postnatal responses may have been less had the mothers had more time to reflect on their experiences [32]. Other researchers will need to consider the optimum timing of before-and-after administration given the context of their own studies.

In addition to the qualitative appraisal, the PaGES Index offers a quantitative comparison to compare the relative importance of the individual’s cited issues before and after. This is illustrated in Tables 1 and 2, S2 and S3.

This is informative both when the issues raised by the participant are the same or similar (in this study, for example, antenatal anticipation of labour pain and postnatal description of the pain experience) or when ‘before’ concerns disappear but are replaced by ‘after’ concerns. In the latter case, for example, while only one of 38 women who had referred antenatally to lack of knowledge about childbirth also cited this postnatally, the concept of ‘Traas’ emerged postnatally. ‘Traas’ is a sentiment in Marathi ranging from mild inconvenience to vexation or even ‘torment’.

While some of the themes identified at both time points were to be expected (‘Pain’, ‘Mode of birth’, ‘Gender of baby’), the PaGES Index allowed the women not only to raise issues like ‘treatment by staff’ and ‘traas’ but to rank their relative importance, giving the researchers an indication of their subjective ‘take’ on their current situation. They could also score their cited areas postnatally to produce an overall index score relating to experience and satisfaction. As has been found in several MGI studies in various countries including India [21, 23, 25, 33], this subjective approach allows for aspects of participants’ lives to be evaluated which standard tools may not cover. For example, variables such as family considerations, notably in relation to the gender of the baby but also in relation to on-going family life and the child’s future education, are not usually included in the assessment of intrapartum outcomes in controlled trials in maternity care [9]. Other factors not covered by the standard tools but recorded as important by the respondents in this study included the gentleness of the practitioner during vaginal examinations, manner of staff and the baby’s future education.

### Coding issues

One challenge in coding is that it anticipates the participants expressing only one priority in each comment. However, comments are not always focused on a single entity: for example, there are two issues to consider in the comment “My husband does not want a caesarean, but I want a healthy baby, whichever way delivery happens”. The baby appears to be the priority here, so the comment was coded as such. Other statements, e.g. “I want my delivery to be safe and normal”, incorporate two equally weighted opinions (safety and normal birth), and so the participant’s overall context must be considered when coding. For example, if the woman has indicated elsewhere that she wishes to have a normal birth, then this particular comment may be coded under ‘safe birth’. Of course, such interpretations must be corroborated by others, not least those on the ground who can confirm or refute cultural assumptions.

### Feasibility of incorporating the PaGES Index within an RCT

Although randomised trials have become increasingly complex and expensive, associated qualitative studies continue to collect data on small sub-samples of 10–20 participants. Data collection typically continues ‘until data saturation’, with the ‘saturation’ referring to the *range* of participant opinions and topics, not to any kind of quantatative assessment for which it is statistically underpowered. So, whilst an alongside qualitative study can provide a rich source of information about the range of issues that some participants have experienced, it cannot say how common these issues and opinions are across the broader population. The brief qualitative assessment within the PaGES Index (using short comments only) is designed to be simple enough that it can be completed within 10 min by all study participants. This provides a statistically powerful way of evaluating the preferences of all study participants, or at least of a statistically meaningful sub-group. It could also be used to compare arms of a comparative study.

An option that we have not explored in this pilot is to amend the data collection form (as with the later MGI studies) so that the participant categorises each comment as ‘positive’ ‘neutral’ or ‘negative’. This approach reduces subjective interpretation by researchers faced with coding and categorising short comments. The bean scores within each code would then be given positive or negative values and summed so as to produce an overall evaluation of the experience and satisfaction of each participant.

### Translation issues

One of the core components of qualitative research is to gain insight into subjective experiences. It is essential to consider language interpretation as part of any cross-culture study due to its influence on the construction of meaning [34]. In order to truly capture the depth and intricacies of an experience, metaphors and narratives are commonly used [35]. These are often language-specific and so do not allow for literal translation or interpretation [36]. Throughout the development process any statements where the meaning was unclear were discussed with researchers based in India. A bilingual translator with a good understanding of the participants’ cultural context is necessary in order to avoid the risk of sentiments being ‘lost in translation’. The participants’ comments are not lengthy, and our experience was that few textual and conceptual translation problems arose. We therefore tentatively suggest that the PaGES Index, with appropriate preparation, support and monitoring, could be used in a wide range of cultural and linguistic contexts. Other tools, by contrast, often require sophisticated and expensive conceptual and linguistic fine-tuning to fit each context in which they are used.

### Acceptability

The qMOLI sub-study was a formal alongside qualitative assessment which included questions on the experiences and perceptions of participants, researchers and clinicians. Respondents reported that completion of the PaGES Index was straightforward, taking just minutes. It provided a platform to build trust between participants and research staff. Most participants indicated that it enabled them to talk freely; they especially liked being asked about their feelings and experiences. Researchers indicated that the PaGES Index was an opportunity to develop an understanding of the women and build relationships with them.

### Strengths and limitations

To our knowledge, no previous RCT concerning induction of labour in a LMIC has included an individualised respondent-driven PREM such as this, although various studies have used health status or specific quality of life instruments. The ‘before-and-after’ aspect allows for direct evaluation of the anticipated and actual impact of the intervention on participants. Using it again very soon after the birth ensured high follow-up rates.

Basing the PaGES Form assessments on the areas of life which the mother considers to be those most important to her avoids the pitfalls of a ‘top-down’ instrument which, however well prepared, may not reflect all the woman’s current concerns [37]. Participant responses may also be too general to identify priority areas or potential solutions easily. The tool asks participants what is important to them but not “What would make things better?”

A tool that, in addition to a numerical comparison of intervention and control groups, also allows for more nuanced analysis of the issues that are important to participants, adds a valuable additional layer of understanding. This could be used to highlight the contextual factors that might influence the success or failure of an intervention. The PaGES Index, like the MGI, can be administered to a large cohort. This raises the prospect of obtaining high-quality qualitative data from all study participants (rather than only the typically small sub-sample included to provide a qualitative add-on to a trial); this can also happen both before and after the intervention. This could help to open up what Sutton calls the ‘black box’ of the mechanisms of effect in such interventions [38].

Preparing the PaGES Index for use in different populations is not time-consuming. The form and its language are simple; even if translation is required this is not a complex task for competent researchers. We do acknowledge that if it is to be used in large sub-samples, then adequate time and other resources must be factored in to collect, clean, and analyse the data, and translate where necessary. All of these processes require appropriately trained / supervised researchers who may not be inexpensive. Nevertheless, the advantage is that this allows for broader population coverage than is possible in a discrete qualitative alongside study.

While the data collected from 68 participants gives us a detailed insight into the participant experience, we cannot say from this pilot dataset what the optimal sampling frame would be for implementing the PaGES Index within other large studies. For example, administering the PaGES Index soon after birth ensured high follow-up rates. It does, however, raise pertinent questions about how time may influence what participants would cite as important and how they might score these issues at different times.

Further research is planned to compare the outcomes of the PaGES Index analysis with the classical alongside qualitative study approach. This will be possible within the MOLI study as both assessments have been undertaken. We will also compare the PaGES outcomes from the two arms of the randomised trial to quantify the difference that the intervention had on the study population.

## Conclusion

We found in this pilot study that it was acceptable, feasible and valuable to ask participants to complete this brief qualitative/quantitative assessment. Hearing what study participants consider to be important to them before and after an intervention provides pertinent and sometimes unexpected insights into what they value. The freeform nature of the PaGES Index should allow it – with suitable planning and application - to be used across cultures, languages and contexts. The coding frame also allows researchers to evaluate the frequency of the different opinions expressed by participants. This significantly strengthens a comprehensive and holistic understanding of the study phenomenon.

The PaGES Index potentially provides a method that can be used across clinical research to summarise and quantify participants’ experiences. Focussing on what participants value should lead to improvements in the quality and impact of research.

### Electronic supplementary material

Below is the link to the electronic supplementary material.

Supplementary Material 1

Supplementary Material 2

Supplementary Material 3

Supplementary Material 4

## Data Availability

The datasets generated and analysed during the current study are not currently publicly available because they are a small part of a larger ongoing study, and the dataset is not locked. When the full dataset analysis is complete the data will be available from the corresponding author on reasonable request.

## List of abbreviations

CI: Confidence Intervals
CRF: Case Report Form
CS: Caesarean Section
IOL: Induction of Labour
LMICs: Low and Middle-Income Countries
MGI: Mother-Generated Index
MOLI: Misoprostol / Oxytocin Labour Induction
Mx: Medication
NVD: Normal Vaginal Delivery
PaGES: Participant-Generated Experience and Satisfaction
PREMS: Patient-Reported Experience Measures
PROM: Patient-Reported Outcomes Measures
RA: Research Assistant
RCT: Randomised Controlled Trial
SD: Standard deviations
Tx: Treatment
