# Supplementary material for: Introducing the participant-generated experience and satisfaction (PaGES) index: a novel, longitudinal mixed-methods evaluation tool
Source: BMC Med Res Methodol. 2023 Sep 28;23:214. doi: 10.1186/s12874-023-02016-1 (PMC10537543; doi:10.1186/s12874-023-02016-1)
Supplement: Supplementary file 1 — Supplementary Material 1 [file 12874_2023_2016_MOESM1_ESM.docx]

**Table S1 Participant characteristics (n=68)**

| **Age** (years) | mean (s.d.) | | 24.3 (3.1) |
| --- | --- | --- | --- |
| **Gestational age** (best estimate in weeks) | mean (s.d.) | | 37.9 (1.9) |
| **Highest completed level of education**  No formal education  Primary  Secondary  Technical / skilled job training  University  Post grad studies | n (%)  n (%)  n (%)  n (%)  n (%)  n (%) | | 1 (1.5)  15 (22.1)  26 (38.2)  0 (0)  22 (32.4)  4 (5.9) |
| **Woman’s employment**  Farming  Professional (teacher, medical, nurse)  Labourer (construction, factory)  Office job  Business (employed, self-employed)  Domestic work (paid)  Housewife  Other | n (%)  n (%)  n (%)  n (%)  n (%)  n (%)  n (%)  n (%) | | 2 (2.9)  0 (0)  0 (0)  2 (2.9)  2 (2.9)  2 (2.9)  60 (88.2)  0 (0) |
| **Parity**  0  1  2 | n (%)  n (%)  n (%) | | 59 (86.8)  8 (11.8)  1 (1.5) |
|  | mean (s.d.) | | 0.2 (0.4) |
| **Gravidity**  1  2  3  4  5 | n (%)  n (%)  n (%)  n (%)  n (%) | | 58 (85.3)  5 (7.4)  3 (4.4)  1 (1.5)  1 (1.5) |
|  | mean (s.d.) | | 1.3 (0.7) |
| **Woman received ≥1 antenatal visits for this pregnancy** | n (%) | | 66 (97.1) |
| **Currently on antihypertensives** | | n (%) | 65 (95.6) |
