# Supplementary material for: Introducing the participant-generated experience and satisfaction (PaGES) index: a novel, longitudinal mixed-methods evaluation tool
Source: BMC Med Res Methodol. 2023 Sep 28;23:214. doi: 10.1186/s12874-023-02016-1 (PMC10537543; doi:10.1186/s12874-023-02016-1)
Supplement: Supplementary file 2 — Supplementary Material 2 [file 12874_2023_2016_MOESM2_ESM.docx]

**Table S2 Overarching Theme 2: Perspectives on the Baby**

| **Antenatal** | | | **Postnatal** | | |
| --- | --- | --- | --- | --- | --- |
| **Overarching concepts** | **Antenatal code** | **To include concepts of** | **Overarching concepts** | **Postnatal code** | **To include concepts of** |
| Baby should "be good" | **Baby is the priority** | The baby is the priority and should be "good" - meaning baby is ok including healthy / safe / well | Baby should "be good" | **Baby is the priority** | Baby is safe and healthy, statements regarding baby’s health |
|  |  |  |  | **Because baby is good, I am good** | Baby is good, AND THEREFORE I am happy / good (relative clause) |
|  |  |  |  | **Concerns / worry about baby pre delivery** | Before delivery, worried / anxious |
|  |  |  |  | **Concerns / worry about baby post delivery** | Now postnatal feels worried / anxious |
| Gender | **Gender of baby** | no preferences, wants girl, wants boy | Gender | **Gender of baby** | had boy / girl, happy gender |
|  | **Family and gender of baby** | ready for any gender, gender preference |  | **Family and gender of baby** | happy had boy / girl |
| Breastfeeding | **Breastfeeding** | concerns / worries / issues with breastfeeding | Breastfeeding | **Breastfeeding** | concerns / worries / issues with breastfeeding |
