# Supplementary material for: Introducing the participant-generated experience and satisfaction (PaGES) index: a novel, longitudinal mixed-methods evaluation tool
Source: BMC Med Res Methodol. 2023 Sep 28;23:214. doi: 10.1186/s12874-023-02016-1 (PMC10537543; doi:10.1186/s12874-023-02016-1)
Supplement: Supplementary file 3 — Supplementary Material 3 [file 12874_2023_2016_MOESM3_ESM.docx]

**Table S3 Overarching Themes 3-6: 3 - Physical Condition; 4 - Psychological Condition;**

**5 - Family; 6 - Looking to the Future**

| **Antenatal** | | | | **Postnatal** | | | |
| --- | --- | --- | --- | --- | --- | --- | --- |
| Overarching themes | Overarching concepts | **Antenatal code** | To include | Overarching themes | Overarching concepts | **Postnatal code** | To include |
| Physical Condition | Blood pressure & Physical Symptoms | **Blood pressure & other physical symptoms** | General comments + concerns about blood pressure including worried, anxious, stressed, tension, symptoms now (dizzy, headache) feel sick | Physical Condition | Blood pressure & Physical Symptoms | **Blood pressure & other physical symptoms** | Current symptoms (dizzy, headache) feeling sick |
| Psychological Condition | Happy | **Happy** | Thoughts of happiness regarding pregnancy, baby, becoming a mother, general comments | Psychological Condition | Happy | **Happy** | General comments of happiness, happy to have a baby, happy to be a mother |
|  | Scared / fear / afraid, Worried / anxious | **Worried / anxious / scared / fear / afraid** | Thoughts of worry, anxiety or fear |  |  |  |  |
|  | No worries | **No worries** | Statements stating a lack of concern or worry |  | No worries | **No worries / difficulty** | statements including no worries or difficulty |
|  |  |  |  |  |  |  |  |
| Family | **Family support** | **Family is good / happy / take care** | Family is good / happy / take care | Family |  | **Family is good / happy / take care** | Family is good / happy / take care |
|  | **Family influences** | ***Family influences*** | Delivery, happy, husband only, other, worried, husband’s job / money, shared family, who will do the work, family will help out with baby |  |  |  |  |
|  |  |  |  |  |  |  |  |
| Looking to the future | Looking to the future | **Looking to the future** | plans to educate well, future education, future life, my baby will not have to endure what I have | Looking to the future | Looking to the future | **Looking to the future** | plans to educate well, future education, future life |
