# Supplementary material for: Introducing the participant-generated experience and satisfaction (PaGES) index: a novel, longitudinal mixed-methods evaluation tool
Source: BMC Med Res Methodol. 2023 Sep 28;23:214. doi: 10.1186/s12874-023-02016-1 (PMC10537543; doi:10.1186/s12874-023-02016-1)
Supplement: Supplementary file 4 — Supplementary Material 4 [file 12874_2023_2016_MOESM4_ESM.docx]

**Additional quantitative analysis**

This file contains four supplementary tables (S4 – S7) with associated explanations.

The total number of times each of the six overarching themes was cited is summarised in Table S5. Overall, each theme was cited less frequently postnatally than antenatally, with the same two exceptions (“perspectives on the baby” and “looking to the future”). However, when only citations attracting an allocation of one or more beans was evaluated, a third theme (“family”) now also occurred more frequently postnatally than antenatally. The differences were all relatively small, but the increase in citation frequency for the “looking to the future” was again statistically highly significant (p<0.001); the difference for the theme “family” also approached statistical significance (p=0.073).

**Table S4 Incidence of zero and non-zero bean score allocations (postnatal vs. antenatal)**

| **Overarching theme:** | **Antenatal** | | | **Postnatal** | | | **% difference (1+ beans) [95% CI]** |
| --- | --- | --- | --- | --- | --- | --- | --- |
|  | **Times cited** | **Beans allocated** | | **Times cited** | **Beans allocated** | |  |
|  |  | **0** | **1+** |  | **0** | **1+** |  |
| **Perspectives on the birth** | 212 | 109 | 103 (48.6%) | 153 | 87 | 66 (43.1%) | -5.4 [-17.1 : 6.3] |
| **Perspectives on the baby** | 92 | 32 | 60 (65.2%) | 132 | 52 | 80 (60.6%) | -4.6 [-18.7 : 9.4] |
| **Physical condition** | 24 | 9 | 15 (62.5%) | 15 | 9 | 6 (40.0%) | -22.5 [-54.6 : 9.6] |
| **Psychological condition** | 53 | 23 | 30 (56.6%) | 46 | 20 | 26 (56.5%) | -0.1 [-22.1 : 22.0] |
| **Family** | 48 | 35 | 13 (27.1%) | 40 | 22 | 18 (45.0%) | 17.9 [ -1.6 : 37.5] |
| **Looking to the future** | 1 | 1 | 0 | 17 | 8 | 9 (52.9%) | 52.9 [ 27.2 : 78.7] |

The mean bean allocation counts for each theme are summarised in Table S6. Including concerns for which no beans were allocated (zero counts), counts fell at the postnatal assessment for three themes (“perspectives on the birth”, “perspectives on the baby”, “physical condition”) and increased for the remaining three themes (“psychological condition”, “family”, “looking to the future”). The differences tended to be numerically small, but those for “perspectives on the birth” and “family” approached statistical significance (p=0.084 and p=0.094 respectively).As expected, when the zero counts were excluded, the mean values all increased considerably. In addition, the standard deviations were now smaller relative to their respective mean values, indicating that the previous slight over-dispersion due to a surfeit of zero values was no longer a problem and that these data were closer to the theoretical Poisson distribution expected with count data. The directions of the differences between the ante- and postnatal assessments remained the same, but now none of the differences even approached statistical significance.

The reasons why mothers allocated no beans to expressed concerns were not explored in this pilot study. It may be reasonable to surmise, however, that this was due to mothers feeling that such concerns, while real and deserving of mention, were relatively minor in magnitude. In which case, Table 4 appears to suggest that the tendency of mothers to report relatively minor concerns varied more between the antenatal and postnatal assessments than the perceived severity of their more major concerns.

**Table S5 Mean (standard deviation) bean scores (postnatal vs. antenatal)**

| **Overarching theme:** | **Antenatal** | **Postnatal** | | **Rate ratio (95% CI)** | |
| --- | --- | --- | --- | --- | --- |
| *All mothers who cited theme (including zero bean citations)* | | | | | |
| **Perspectives on the birth** | 2.78 (4.21) | 2.25 (3.40) | | 0.807 (0.632 : 1.030) | |
| **Perspectives on the baby** | 4.92 (5.09) | 4.28 (4.84) | | 0.869 (0.674 : 1.122) | |
| **Physical condition** | 3.46 (3.59) | 1.67 (2.77) | | 0.482 (0.186 : 1.252) | |
| **Psychological condition** | 3.49 (4.91) | 4.33 (5.17) | | 1.239 (0.768 : 1.999) | |
| **Family** | 1.00 (2.07) | 1.85 (2.67) | | 1.850 (0.901 : 3.797) | |
| **Looking to the future** | 0 | 3.12 (3.84) | | not calculable | |
| *Only mothers who cited theme and allocated one or more beans* | | | | | |
| **Perspectives on the birth** | 5.74 (4.43) | | 5.21 (3.37) | | 0.908 (0.732 : 1.127) |
| **Perspectives on the baby** | 7.55 (4.45) | | 7.06 (4.36) | | 0.935 (0.729 : 1.200) |
| **Physical condition** | 5.53 (2.97) | | 4.17 (2.99) | | 0.753 (0.413 : 1.374) |
| **Psychological condition** | 6.17 (5.11) | | 7.65 (4.66) | | 1.241 (0.878 : 1.754) |
| **Family** | 3.69 (2.46) | | 4.11 (2.54) | | 1.113 (0.715 : 1.735) |
| **Looking to the future** | 0 | | 5.89 (3.33) | | Not calculable |

The mean postnatal satisfaction scores for each theme are summarised in Table S7. In general terms, satisfaction levels were high and significantly correlated with bean allocations. The two exceptions were the themes “ “perspectives on the birth” and “physical” for which, on average, satisfaction levels tended to be neutral and did not correlate with satisfaction scores. These findings differed only marginally when zero bean scores were included or excluded.

**Table S6 Satisfaction scores and their relationships with bean allocations**

| **Overarching theme:** | **Satisfaction score:** | | **Mean increase in satisfaction score (95% CI) per additional bean allocations:** | | |
| --- | --- | --- | --- | --- | --- |
|  | **mean** | **s.d.** | **1 bean increase** | **5 beans increase** | **p-value** |
| *All mothers who cited theme (including zero bean citations)* | | | | | |
| **Perspectives on the birth** | 5.49 | 3.25 | 0.14 (-0.08 : 0.35) | 0.68 (-0.39 : 1.74) | 0.214 |
| **Perspectives on the baby** | 8.23 | 2.52 | 0.06 ( 0.01 : 0.12) | 0.32 ( 0.03 : 0.61) | 0.033 |
| **Physical condition** | 4.67 | 3.62 | 0.31 (-0.28 : 0.90) | 1.55 (-1.42 : 4.52) | 0.305 |
| **Psychological condition** | 8.89 | 1.71 | 0.11 ( 0.04 : 0.19) | 0.57 ( 0.19 : 0.96) | 0.003 |
| **Family** | 8.46 | 2.02 | 0.20 ( 0.00 : 0.39) | 0.98 ( 0.01 : 1.95) | 0.047 |
| **Looking to the future** | 9.00 | 2.06 | 0.16 (-0.02 : 0.34) | 0.81 (-0.08 : 1.69) | 0.073 |
| *Only mothers who cited theme and allocated one or more beans* | | | | | |
| **Perspectives on the birth** | 6.20 | 3.35 | -0.01 (-0.36 : 0.33) | -0.06 (-1.79 : 1.67) | 0.946 |
| **Perspectives on the baby** | 8.17 | 2.70 | 0.15 ( 0.01 : 0.22) | 0.75 ( 0.06 : 1.45) | 0.034 |
| **Physical condition** | 4.83 | 4.31 | 0.65 (-0.08 : 1.38) | 3.25 (-0.42 : 6.92) | 0.082 |
| **Psychological condition** | 9.19 | 1.41 | 0.14 ( 0.02 : 0.26) | 0.70 ( 0.11 : 1.29) | 0.020 |
| **Family** | 8.89 | 1.57 | 0.20 (-0.08 : 0.40) | 1.01 (-0.42 : 2.45) | 0.166 |
| **Looking to the future** | 9.00 | 2.29 | 0.43 (-0.06 : 0.91) | 2.14 (-0.29 : 4.57) | 0.084 |

The mean satisfaction and hybrid satisfaction*bean count scores for the mothers randomised into the two MOLI study treatment groups are summarised in Table S8. Given the small numbers of observations available for analysis, the absence of group differences that were statistically significant at the conventional 5% level is not surprising. However, encouragingly, some differences approached significance, indicating that, with proper consideration of statistical power and sample sizes, both scales will be sufficiently sensitive to detect clinically important differences in satisfaction between patient groups. On balance, the hybrid score was slightly better in this respect than the satisfaction scale on its own, but possibly only marginally so.

**Table S7 Mean (SD) satisfaction scores by treatment group**

| **Overarching theme:** | **Satisfaction scores** | | | **Log(Satisfaction * beans) scores** | | |
| --- | --- | --- | --- | --- | --- | --- |
|  | **Group 1** | **Group 2** | **Difference (95% CI) [p]** | **Group 1** | **Group 2** | **Difference (95% CI) [p]** |
| **Perspectives on the birth** | 6.54 (2.92) | 5.72 (3.25) | -0.81 (-2.47 : 0.85) [0.336] | 2.72 (1.19) | 2.31 (0.93) | -0.41 (-1.00 : 0.17) [0.168] |
| **Perspectives on the baby** | 8.32 (2.73) | 7.42 (2.91) | -0.90 (-2.65 : 0.84) [0.309] | 2.98 (1.14) | 3.14 (1.21) | 0.17 (-0.25 : 0.59) [0.428] |
| **Physical condition** | 7.17 (2.23) | 3.60 (4.10) | -3.57 (-7.34 : 0.21) [0.064] | 2.69 (1.13) | 1.62 (0.80) | -1.07 (-2.25 : 0.10) [0.074] |
| **Psychological condition** | 9.80 (0.45) | 8.80 (1.86) | -1.00 (-2.38 : 0.38) [0.155] | 4.21 (1.21) | 3.10 (1.04) | -1.11 (-2.21 : -0.01) [0.049] |
| **Family** | 8.80 (1.79) | 8.42 (2.19) | -0.38 (-2.20 : 1.43) [0.679] | 2.71 (0.82) | 2.85 (0.97) | 0.14 (-0.73 : 1.00) [0.756] |
| **Looking to the future** | Too few observations for a sensible formal analysis. | | | | | |
